# Traumatic Encephalocele in a 16-Year-Old Male: A Rare Phenomenon

**DOI:** 10.7759/cureus.44507

**Published:** 2023-09-01

**Authors:** Rajul Oswal, Deep Hathi, Hemant R Shah, Geetanjali Joshi, Rinkle Gemnani

**Affiliations:** 1 Department of Radiology, Civil Hospital, Silvassa, IND; 2 Department of Endocrinology, Nil Ratan Sircar Medical College and Hospital, Kolkata, IND; 3 Department of Radiology, Employees' State Insurance Corporation (ESIC) Hospital, Delhi, IND; 4 Department of Medicine, Jawaharlal Nehru Medical College, Wardha, IND

**Keywords:** case report, trauma, csf leak, fronto-ethmoidal, encephalocele, computed tomography

## Abstract

The term "encephalocele" refers to the herniation of brain tissue caused by a cranial bone defect. It could be congenital, traumatic, neoplastic, or arise spontaneously. The possibility of traumatic fronto-ethmoidal encephalocele should be considered in patients who have experienced trauma. We report a case of a 16-year-old male with a recent history of a bike accident presented with sudden unilateral rhinorrhea. Non-contrast computed tomography (NCCT) brain was done, which showed findings of left fronto-ethmoidal encephalocele. The patient was managed with single-staged surgery without any complications.

## Introduction

An encephalocele is defined as the herniation of brain tissue through a skullbone defect, which can be congenital, traumatic, neoplastic, or spontaneous in origin. Traumatic encephalocele usually results secondary to head trauma or can be iatrogenic [1]. Almost 96% of all cases of acquired encephalocele are traumatic in origin. Very few cases of traumatic encephalocele have been reported, with several cases around 25 in a study published in 2010 [2]. Encephalocele, if left undiagnosed, can result in potentially life-threatening complications, like meningitis and cerebrospinal fluid (CSF) leakage; thus, early diagnosis of this condition can prevent such complications [3]. We hereby present a case of traumatic fronto-ethmoidal encephalocele.

## Case presentation

A 16-year-old male presented to the casualty with an episode of loss of consciousness for five minutes with spontaneous recovery and no similar previous episodes. He had no significant past medical history, and he was not on any medications. His family history was unremarkable. He had a past history of road traffic accidents in the form of a bike accident a month ago. He was traveling at a medium speed (40-50km/hour) without wearing a helmet when he met with an accident with a car and fell on the road with mild trauma to the frontal area in the form of bruises and mild swelling over the forehead which was managed at home. The patient did not seek any medical assistance for the same. During the present visit, apart from the loss of consciousness for five minutes in the morning as described above, the patient only complained of a unilateral running nose, which he noticed two days ago and was sudden in onset, whitish in color, non-foul smelling, and not associated with blood stain. There was no history of fever, neck pain, weakness in both limbs, or sensory symptoms in the form of tingling or numbness.

**Figure 1:**
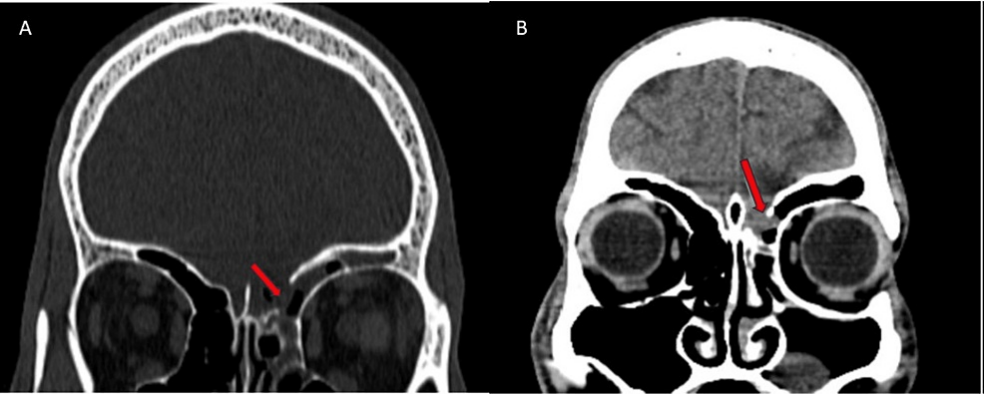
Defect in a vertical plate of left cribriform bone as shown by the red arrow (A), herniation of the left frontal lobe through the bony defect as shown by the red arrow (B)

On physical examination, the patient was conscious and alert, with a Glasgow Coma Scale (GCS) score of 15/15. Neurological examination shows the power of 5/5 in all four limbs and no neuro-deficit. Other systemic examinations were unremarkable. Routine blood investigations like complete blood count, liver function test, and kidney function test were done, which were within normal limits. The patient underwent non-contrast CT brain, which showed a linear displaced fracture of the posterior wall of the left frontal sinus and the vertical portion of the left cribriform plate along with herniation of left frontal lobe parenchyma through this defect, as shown in Figure 1, confirming the diagnosis of left fronto-ethmoidal encephalocele. β-2 transferrin test was done for this patient from nasal discharge, which was significantly raised at 2560 mg/dl (normal range- 170-370 mg/dl), further supporting our diagnosis. The patient was referred to a higher dedicated neuro-interventional center where he underwent single-staged excision of the encephalocele and correction of craniofacial deformity by a team consisting of a neurosurgeon and maxillofacial surgeon. The whole surgery was uneventful, and the patient did not have any post-operative complications.

## Discussion

Anatomically, the ethmoid sinus is surrounded by thin walls and hence can be easily damaged by trauma and can result in encephalocele [4]. Cerebrospinal fluid (CSF) leak can occur anytime following trauma, ranging from a few hours to months [5], and meningitis usually within the first three months after trauma [6]. In our case, luckily our patient did not have any symptoms of meningitis and underwent early surgical intervention. According to current practice to confirm CSF leak, β-2 transferrin level is detected in fluid [7]. In our case, unilateral rhinorrhea was suspected to arise from CSF; hence, β-2 transferrin was advised, and the diagnosis was confirmed. Imaging modalities like thin-section CT, CT cisternography, MR, MR cisternography, and radionuclide cisternography are currently used to detect fistula in patients with CSF leaks [6]. In our patient, only CT images were taken due to financial constraints of patient. Due to wide bony defects, CT was sufficient to detect encephalocele in our patient.

Encephaloceles are classified according to the anatomical area of the bony defect, which is important from a surgical point of view. Recently, a revised classification by Gerhardt includes head dome, occipital, fronto-ethmoidal, and basal encephalocele [8]. In our patient, a fronto-ethmoidal encephalocele was observed, which was extending into the frontal and ethmoidal sinus. Management is usually driven by the cranial fracture type and accompanying CSF leak severity. In the case of the encephalocele and CSF fistula, surgical management in the form of removing the herniated mass and reconstruction of cranial deformity is preferred [2]. This can be achieved by two main techniques; first is combined intra- and extra-cranial/ anterior nasal incision and second is pure extra-cranial approach/anterior facial approach. Some institutes prefer two-staged procedures where the first stage is performed by a neurosurgeon, which involves the removal of encephalocele by craniotomy, and the second stage is performed by the maxillofacial surgeon, which deals with the correction of craniofacial deformities [2]. In our case, the patient had encephalocele and CSF leak, and the patient underwent a single staged procedure with no anterior facial exposure.

If this is left untreated, it may lead to permanent CSF fistulas, meningitis, and intractable seizures [9,10].

## Conclusions

In a case of post-traumatic unilateral rhinorrhoea, fronto- ethmoidal encephalocele should be considered as a differential. Prompt diagnosis of this condition is beneficial as it could prevent several life-threatening complications like CSF leak and meningitis. It is possible to diagnose encephalocele and CSF leak using radiological modalities like CT and MRI along with clinical symptoms and hence should be screened if history is suggestive.
